# Successful restoration of archived ovine formalin fixed paraffin-embedded tissue DNA and single nucleotide polymorphism analysis

**DOI:** 10.1007/s11259-022-09937-0

**Published:** 2022-05-26

**Authors:** Amanda Kravitz, Ron Tyler, B. Murali Manohar, B. Samuel Masilamoni Ronald, Michael T. Collins, Nammalwar Sriranganathan

**Affiliations:** 1 Center for One Health Research, Department of Biomedical Sciences and Pathobiology, Virginia-Maryland College of Veterinary Medicine, Virginia Polytechnic Institute and State University, Blacksburg, VA USA; 2 Tamilnadu Veterinary and Animal Sciences University, Madhavaram Milk Colony, Chennai, 600051 Tamil Nadu India; 3 Department of Pathobiological Sciences, University of Wisconsin-Madison School of Veterinary Medicine, Madison, WI USA

**Keywords:** SNP analysis, Sheep, FFPE tissues, FFPE DNA restoration, Illumina Ovine SNP50 BeadChip

## Abstract

Archived formalin fixed paraffin-embedded (FFPE) tissues are powerful tools in medicine, capable of harboring diagnostic and genetic answers to challenging clinical questions. Successful utilization of DNA derived from FFPE samples is dependent upon repairing DNA damage generated from the fixation process. Methods to repair FFPE DNA have been successful in human medicine for a variety of research and clinical applications, yet remain underutilized in veterinary medicine. Despite the available technology, our study is the first to evaluate the repair of FFPE derived DNA from veterinary species for single-nucleotide polymorphism (SNP) analysis using the Illumina OvineSNP50 BeadChip and Illumina FFPE QC and DNA Restore kit. To accomplish this, 48 ovine FFPE samples were run using the Illumina OvineSNP50 BeadChip with and without restoration. Compared to pre-restore data, we found increased sample call rates, SNP call frequency, and assay metrics for all samples post-restoration. Further, we utilized four sheep with available parallel fresh DNA and FFPE DNA to compare assay metrics and genotype calls between the two starting sample types. Although fresh samples generated increased call rates, we found 99% concordance in allele calls between restored FFPE and fresh DNA for all four samples. Our results indicate successful restoration and genotyping of ovine FFPE samples using this technology, with potential for utilization in other veterinary species.

**Supplementary Information:**

The online version contains supplementary material available at 10.1007/s11259-022-09937-0.

## Introduction

In human medicine formalin fixed paraffin-embedded (FFPE) samples have allowed for biomarker discovery, retrospective genetic association studies, and identification of common mutations found in various cancers (Oosting et al. 2007; Guo et al. 2019). Only recently has this sample type been utilized in veterinary medicine via a genome-wide gene expression analysis on FFPE tumor samples from canine oral malignant melanoma (Bowlt Blacklock et al. 2019). The increased validation and understanding of the human genome compared to veterinary species, and the FFPE mediated DNA damage that complicates downstream analysis have likely contributed to the limited application in veterinary medicine comparatively. Fixation of tissues can result in increased fragmentation of DNA, formaldehyde induced crosslinks, and deamination of cytosine bases which complicates downstream molecular analysis (Do and Dobrovic 2014). Technologies developed for use on human FFPE DNA exist to repair samples in preparation for use on commercially available SNP arrays such as Ilumina BeadChip products (Adler et al. 2013). These methods were developed for human samples and utilize human non-specific primers to successfully restore human FFPE DNA for use on SNP arrays. Studies validating the restoration of human FFPE DNA for SNP analysis using Illumina’s FFPE QC and Restore kit have resulted in increased SNP data quality compared to unrestored samples (Jasmine et al. 2012; Hosein et al. 2013). The majority of these studies investigated the concordance between matched frozen fresh (FF) and FFPE DNA samples in a variety of human oncology research to identify SNP tumor variants (Lips et al. 2005; Harada et al. 2011; Jasmine et al. 2012; Hosein et al. 2013; Hertz et al. 2015). Despite validation in human FFPE samples, to the best of our knowledge the efficacy of FFPE DNA restoration on livestock samples and downstream SNP array analysis has yet to be investigated.

Livestock breeding and preventative health programs have recently placed increased importance on genetic research to generate selective breeding programs. These programs aim to enhance production traits, tolerance to extreme environmental conditions, and tolerance/resistance to infectious disease (Rexroad et al. 2019). Commercial single nucleotide polymorphism (SNP) arrays created for livestock species have allowed for a low-cost, genome-wide interrogation of thousands of SNP markers in multiple individuals simultaneously. This technology has allowed for increased genotyping of individual animals, and consequently identification of SNPs and candidate genes associated with traits of interest for selective breeding programs. Data obtained from species-specific SNP arrays has enabled comparative population structure analyses (Kijas et al. 2012; Rothschild et al. 2017), identification of genetic markers associated with production traits including wool and meat quality (Brito et al. 2017a; Dovc et al. 2019), as well as genome-wide association studies (GWAS) investigating host resistance to infectious diseases (Vieira Benavides et al. 2015; Moioli et al. 2016). This technology in combination with open-source databases including PubMed dbSNP, and the Sheep HapMap project, can further refine and contribute to previously identified SNPs associated with specific traits. Selective breeding programs in the New Zealand sheep industry have successfully utilized this technology resulting in increased profitability, performance, and generation of composite breeds for variable climate tolerance (Brito et al. 2017b). The application of SNP microarrays in identification of livestock disease tolerance/resistance markers would be enhanced if FFPE samples could be restored and utilized Obtaining sufficient numbers of cases and controls with specific exposure and diagnostic data is challenging in veterinary medicine, and the vast archives of FFPE samples may help fill this gap. FFPE samples also remain stable at room temperature for years, allowing for ease of sample sharing from various breeds, institutions, and countries compared to fresh tissue (Gnanapragasam 2010; Yun et al. 2018).

Here, we evaluate the utility of the Illumina FFPE QC and FFPE Restore kit protocols on 48 FFPE sheep samples used for SNP analysis using Illumina’s OvineSNP50 BeadChip. Assay metrics used to evaluate restoration and overall results were based on Illumina product sheets, including sample call rate, distribution of GenCall scores assigned to each called genotype (sample 10pGC), SNP call frequency, and average minor allele frequency (MAF). Assay metrics were compared for 48 FFPE sheep samples pre and post-restoration, then further compared allele calls between four matched FFPE and fresh DNA to determine if restored FFPE samples were capable of successful genotyping. To our knowledge, this is the first report evaluating the ability of this restoration technology using sheep FFPE samples for downstream SNP analysis. Our evaluation of this technology using animal samples aims to determine if these protocols can be used for archived FFPE tissues in veterinary medicine applications, including selective breeding and disease resistance research.

## Materials and methods

### Samples

A total of 48 ovine FFPE samples were obtained from a total of 5 flocks, where breed, age of animal, and age of FFPE sample varied depending upon available information see Supplementary Table 1 for details. Three archived samples were gifted from Dr. Brodersen at University of Nebraska, three samples from Dr. Kim, University of Mississippi, and three samples gifted by Dr. Ramachandran from Oklahoma State University. Four samples provided with the aid of Dr. Michael Collins of University of Wisconsin-Madison had parallel FFPE and whole blood samples available, and were used to compare SNP assay results and sample type. The remaining 35 FFPE samples originated from Kattupakkam sheep from Tamil Nadu Veterinary and Animal Sciences University (TANUVAS) in Chennai India. Regardless of origin, all FFPE samples were fixed in 10% buffered formalin and cassette’s and cut scrolls held at room temperature (15–25 °C) until used for DNA extraction. The four whole blood samples were received on ice and processed the same day.

### DNA extraction

FFPE DNA extractions were done following the Qiagen FFPE DNA extraction kit protocol starting with (25 mg) of FFPE tissue per sample. First, xylene (1 mL) was added to each sample and vortexed 10 s to remove paraffin wax. Tubes were then centrifuged at (16,100 × g) for 2 min to obtain a pellet, and supernatant carefully removed. 100% ethanol (1 mL) was added to the pellet and vortexed for 10 s to mix, then centrifuged at (16,100 × g) for 2 min. Supernatant was carefully removed, and tubes incubated at 37 °C until ethanol was evaporated and pellet appeared dry. Pellet was then resuspended in ATL buffer (180 μL) and Qiagen Proteinase K (20 μL of 20 mg/mL stock) and vortexed for 10 s to mix. Samples were then incubated in a 56 °C water bath until tissues were completely lysed, between 8–12 h. Lysed samples were then incubated at 90 °C for 1 h, followed by addition of AL buffer (200 μL) and 100% ethanol (200 μL). The resulting lysate was transferred to provided (2 mL) spin columns and spun for 1 min at (6,000 × g), and flow through discarded. Wash buffer AW1 was added (500μL) to tubes and spun at (6,000 × g) for 1 min and flow through discarded. Wash buffer AW2 was then added (500μL) and again spun at (6,000 × g) for 1 min and flow through discarded. The wash steps were then followed by centrifugation at (16,100 × g) for 3 min to dry membrane. Lastly, extracted FFPE DNA was eluted using kit provided ATE buffer (50 μL) per sample.

Four whole blood samples with matching FFPE tissues from the same sheep were shipped in EDTA tubes on ice overnight, and processed the following upon arrival. Whole blood was diluted (1:2) in sterile phosphate buffered saline (PBS) with 2% fetal bovine serum (FBS) and layered onto peripheral blood mononuclear cell (PBMC) isolation tubes (StemCell Technologies Inc) with density gradient media (4 mL). Tubes were spun (1,200 × g) for 10 min and PBMCs isolated, and washed twice in PBS with 2% FBS (4 mL). Washed cells were spun (300 × g) for 6 min, cell pellet was obtained and resuspended in PBS (200 μL) and Qiagen Proteinase K (20 μL of 20 mg/mL stock). Cell suspension was used for genomic DNA extraction using the Qiagen QiaAMP Blood and Tissue Mini Kit following kit instructions. Fresh DNA was eluted with AE buffer (100 μL).

For both FFPE and fresh DNA extracted, concentration (ng/μL), 260/280 and 260/230 ratios were measured using the Nanodrop ND-100 Spectrophotometer and DNA held at -20 °C until use.

### Ilumina FFPE QC

To determine if ovine FFPE DNA is compatible with Illumina’s HD FFPE Restore Protocol, samples must pass the FFPE QC assay protocol. Briefly, a qPCR reaction using Illumina human universal primers was run in triplicate using (4μL) of a diluted (1 ng/μL) DNA solution for each sample. Non template controls of molecular grade water, and 1:100 diluted QCT_ST kit control was used with Sigma 2X qPCR master mix (Syber Green Sigma Inc.) and run on a BioRad iCycler. Reaction volumes (20 μL) were subjected to an initial activation step at 95 °C for 10 min followed by 40 cycles of 95 °C for 30 s, 57 °C for 30 s, and 72 °C for the final 30 s per the Illumina HD FFPE QC assay protocol. Delta Cq values were obtained by subtracting the average Cq value from QCT_ST control from the average Cq for each sample. FFPE samples with Delta Cq values of less than 5 were deemed usable for the FFPE Restore protocol as per Illumina kit instructions.

### Illumina FFPE restoration and HD Infinium OvineSNP50 BeadChip

48 FFPE DNA samples were run on Illumina’s HD Infinium OvineSNP50 BeadChip twice, once pre-restoration and once post-restoration. Restoration protocol and all Infinium BeadChips were run at the Mammalian Genotyping Core at University of North Carolina at Chapel Hill using Illumina iScan technology. The 53,516 SNPs included on the assay align with the publicly available Oar_v4.0 *Ovis aries* genome assembly submitted by the International Sheep Genomics Consortium (ISGC). The same 48 FFPE samples pre-and post-restore were run separately on different chips to reduce data noise and evaluate effect of restoration on SNP assay metrics. For FFPE restoration, samples passing FFPE QC were subject to Ilumina FFPE Restoration Kit protocol using (100 ng) of FFPE DNA per sample. Multiple incubation steps using proprietary Illumina buffers and technology induced two enzymatic reactions to restore FFPE mediated DNA damage for subsequent use on Illumina BeadChips. The restoration workflow product was then run on the OvineSNP50 BeadChip to obtain post-restore SNP results.

The four available matched fresh samples, were run by the same technologist and Illumina OvineSNP50 BeadChip but separately to decrease variation due to sample type. These fresh samples were run in a group of 336 for a previous project, with four matching FFPE samples which allowed for direct comparison between sample types on the same individual. The remaining 331 samples were not directly compared as no matching FFPE samples were available. The assay metrics from the four sheep with matching FFPE (pre and post restore) and fresh DNA samples were compared to determine if restoration was successful and allowed for accurate SNP analysis.

### Data analysis

Illumina FFPE QC-qPCR data was analyzed using BioRad iCycler software (BioRad Inc.) where average delta QC values calculated per FFPE DNA sample. Passing samples were submitted to the Illumina FFPE Restore kit and OvineSNP50 BeadChip where raw intensity files and product specific controls were analyzed using Illumina GenomeStudio software. Pre-restore, post-restore and fresh samples were analyzed in separate GenomeStudio projects where genotypes were called according to Illumina cluster algorithm. Overall sample call rates, SNP call frequency and average MAF values were compared between pre-and post-restore FFPE assays to determine if restoration was successful.

To assess assay performance, post-restore FFPE data and fresh data were separately analyzed using Illumina’s GenomeStudio cluster algorithm. Poor performing SNPs and samples were removed to generate a pool of successfully genotyped SNPs for comparison. FFPE samples with call rates less than 80%, and outliers were also removed. SNPs with call frequency less than 80%, and SNPs with MAF less than 1% were also removed from FFPE data set. Fresh sample metrics varied due to higher quality DNA, where samples with call rates of less than 95% were removed in addition to outliers. SNPs from fresh samples were removed if call frequency was less than 95% and MAF less than 1%. Remaining SNPs and samples were used for further analysis.

Percent concordance between post-restore and fresh sample types was calculated using data from the four individuals with overlapping samples. Post-restore FFPE allele calls were exported and subsequently imported to align with fresh DNA assay data. The GenomeStudio software (Illumina Inc.) compared genotypes called for the overlapping samples between the projects and identified concordant (1), discordant (0), and non-overlapping or no-call SNPs (-1). The software then calculated the percentage concordant SNPs across sample types for the same individual, resulting in 0–100% of matched allele calls between the two projects.

## Results

### FFPE QC-qPCR

The Illumina FFPE QC step protocol was followed to determine if our FFPE samples were capable of being restored using Illumina technology. Samples with delta QC value of (< 5) are capable of being repaired to then be used on the array. All 48 of our samples passed this cutoff with an average delta QC of (-16.94), and a range of (-20.18) to (-14.95). These values were consistent across all three replicates per sample. These results are well within the Illumina cutoff of delta QC (< 5) indicating all samples could be run using the restore protocol.

### Influence of FFPE DNA restoration on OvineSNP50 assay metrics

**Fig. 1:**
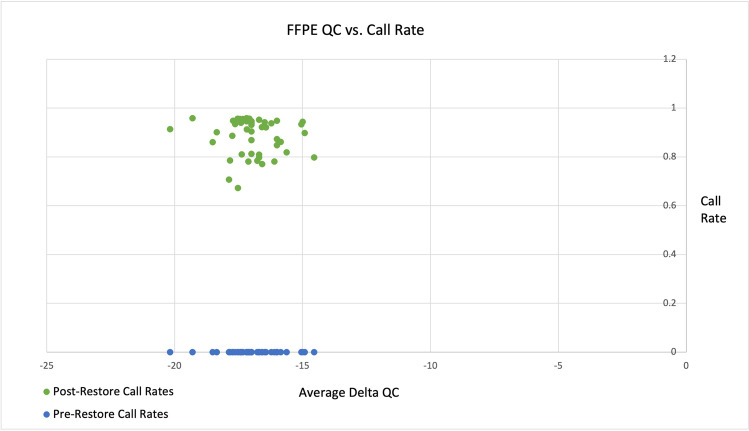
FFPE QC vs. Call Rate

We were able to directly compare the influence of the FFPE restore step using 48 archived samples with and without the restoration step. First, the FFPE QC values for pre and post restore call rates were evaluated and found a substantial increase in call rates for all 48 samples (Fig. 1:FFPE QC vs. Call Rate). Pre-restore sample call rates were zero for all 48 samples, indicating that the degree of DNA damage from these non-restored FFPE samples was too extensive for SNP analysis. Pre-restore SNP average call frequency and GenTrain scores were both low at (0.2616 and 0.2294) respectively. Together, our results indicate unsuccessful genotyping with the Illumina OvineSNP50 Beadchip on FFPE samples without restoration.

Sample call rates are plotted against the average delta QC value obtained by the Illumina FFPE QC kit for all 48 samples pre and post-restoration using GenomeStudio software. Delta QC values of < 5 are considered to be usable for the subsequent restoration step. Here it is evident that pre-restore all samples had call rates of 0, where the post-restore samples had increased call rates with an average of 0.8856*.*

Upon evaluating the post-restore sample metrics, significant changes in assay metrics were found indicative of successful restoration and use on the SNP chip assay. All post-restore samples had increased sample call rates with an average of (0.8856) and a range of (0.6726–0.9581) compared to zero for all samples pre-restore. Other Illumina quality metrics including SNP call frequency, and GenTrain score were also significantly enhanced, with an average of (0.8856) and (0.6738) respectively. Evaluated metrics in pre-FFPE restore and post-FFPE restore can be found in Table 1.

**Table 1:** Pre vs. Post- Restore FFPE Assay Metrics

Assay Metrics	Pre-FFPE Restore	Post-FFPE Restore
Sample Call Rate	0	0.88
MAF	0.09	0.25
SNP Call Frequency	0.26	0.88
GenTrain Score	0.23	0.67

All values were calculated from column statistics using GenomeStudio Software, and mean values calculated in Excel (Microsoft inc.). All 48 FFPE samples and 53,516 SNPs were included in the analysis. Sample call rate and SNP call frequency was calculated by number of calls/(no calls + calls and Minor allele frequency (MAF) reports the average frequency of minor alleles across all loci. The GenTrain score is a quality metric indicative of reliability of a genotype call across all SNPs, where 1 is indicative of high reliability and 0 is low reliability based on GenomeStudio clustering algorithm*.*

### Percent concordance restored FFPE vs. Fresh DNA

**Fig. 2:**
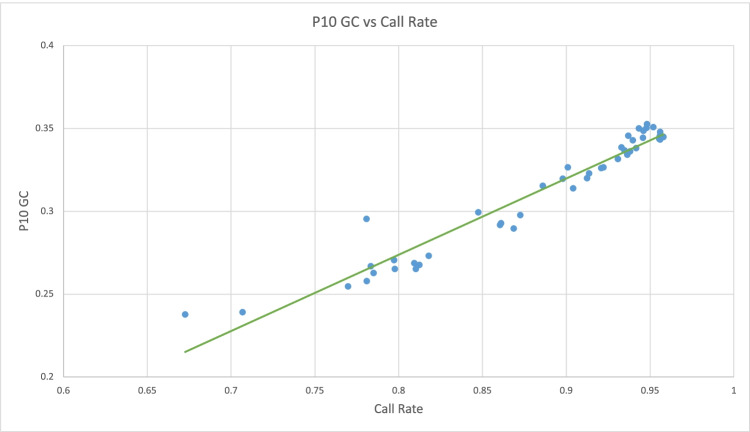
p10GC vs. Call Rate

**Fig. 3:**
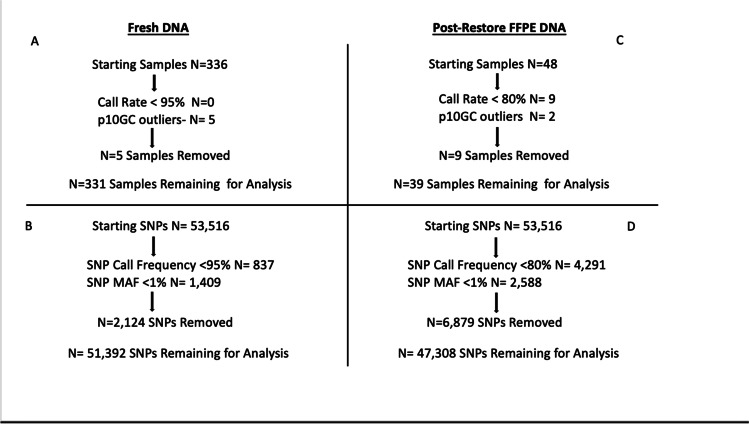
Legend: SNP and Sample Inclusion Criteria

To compare results between post-restore FFPE samples and parallel fresh DNA, both data sets were run and analyzed in separate GenomeStudio projects. To identify poor performing samples and outliers, the post-restore p10GC rate vs. post-restore call rates were plotted as advised by Illumina. **(**Fig. 2: p10 GC vs. Call Rate). From the 48 FFPE samples, nine were removed with call rates lower than 0.80, with two outlier samples with call rates of 0.67 and 0.707 (OKST1 and C696 respectively). SNP and sample cutoff metrics and values can be seen in (Fig. 3: SNP and Sample Inclusion Criteria), where post-restore FFPE and fresh samples and SNPs were pruned using values previously reported by sample type (Hertz et al. 2015; Brito et al. 2017b; Dovc et al. 2019). After removing poor performing samples and SNPs, a total of (47,308) successfully genotyped SNPs from 39 post-restore FFPE samples were used for analysis. Four FFPE samples had parallel fresh DNA available that was run as part of a separate project (unpublished data). After data pruning, (51,392) SNPs from 331 sheep including the four matched samples were available for analysis, which allowed comparison of only quality SNPs from each sample type.

To identify outlier samples to exclude for future analysis, Illumina recommends to plot the 10% GC rate vs. post-restore call rates. 10% GC score is a quality metric for samples representative of the 10^th^ percentile of distribution of GenCall scores across all called genotypes for a given sample. Nine total samples were found to have call rates lower than our call rate cutoff of 0.80. Two outlier samples were among the nine below the cutoff, all 9 samples were removed. Linear trend line is in green. 

Overall schematic for sample and SNP inclusion criteria for both FFPE and fresh DNA samples. FFPE and fresh DNA were analyzed in separate GenomeStudio projects. Different metrics for Fresh and post-restore FFPE data were used to align with variation in DNA quality. Panels **A** and **B** represent Fresh DNA metrics and panels **C** and **D** are for post-restore FFPE results: (**A**) Fresh DNA sample criteria is shown where 5 samples failing to meet inclusion criteria were removed. (**B**) Fresh DNA SNP criteria is shown where a total of 51,392 SNPs were included, and 2,124 SNPs were removed. (**C**) A total of 9 Post-restore FFPE samples failed and were removed, leaving 39 samples passing. (**D**) Post-restore SNP metrics editing resulted in a total of 47,308 SNPs for analysis.

Passing samples and SNPs were included in calculating percent concordance between the two data sets. Allele calls from the post-restore FFPE project were exported and imported into the fresh sample project, allowing the GenomeStudio software to compare genotypes for the matching four sheep between sample types. We found concordance rates of (99%) for each of the overlapping samples, indicating successful genotyping of restored FFPE samples using these methods. Direct comparison of sample type influence on call rates for the overlapping samples and percent concordance can be seen in Table 2.

**Table 2:** Sample Type (FFPE vs. Fresh) Effect on Call Rate

Sample ID	Pre-Restore FFPE Call Rate	Post-FFPE Call Rate	Fresh Call Rate	% Concordance
1741	0	0.95	0.99	0.995
J-24	0	0.95	0.99	0.995
15111	0	0.95	0.99	0.995
4106	0	0.95	0.99	0.996

All four samples were run using the FFPE restore protocol, and freshly isolated DNA for SNP50 analysis. Values generated in GenomeStudio (Illumina Inc). Data from Pre-Restore FFPE call rate, Post-FFPE, and Fresh Call rate shows comparison for the same four sheep using three different workflows and two different starting sample types. Percent Concordance was calculated using GenomeStudio by comparing allele calls per included SNP after data pruning between post-restore FFPE and fresh samples shown here.

## Discussion

To the best of our knowledge, this report is the first to test the Illumina repair protocol on non-human FFPE DNA for subsequent use on the OvineSNP50 BeadChip. This proprietary technology has been applied in human medicine for years (Gnanapragasam 2010; Harada et al. 2011; Hosein et al. 2013), but has yet to be utilized widely in veterinary medicine. The restore protocol tested here employs a combination of enzymatic incubation steps to ligate the highly fragmented FFPE DNA, resulting in double-stranded DNA capable of genotyping using Illumina SNP chips. The restore step and QC-qPCR protocols were generated for human samples, and utilizes universal human primers for QC. It was unknown if the QC or restore protocols would be successful on archived FFPE sheep samples, as Illumina has not conducted non-human FFPE assay restoration trials and thus species specific cutoff metrics do not exist. Here, we tested the ability of the FFPE restore and QC protocols to predict if samples can be successfully restored, and genotype concordance compared to fresh sample DNA.

Our results from the QC-qPCR runs indicated that all 48 of the FFPE samples were capable of being restored according to Illumina’s cutoff of < 5. Our samples resulted in very low negative values for all samples with an average of (-16.94) across triplicates for each. These values are much lower than previously reported, which could be due to the differences in qPCR machines used, lack of species-specific cutoff values and validation, or due to a differential interaction between the human universal primers included in this protocol with our sheep samples (Jasmine et al. 2012; Hosein et al. 2013). Compared to Illumina’s FFPE DNA Analysis Data Sheet (Illumina), which found (35% to 95%) of human FFPE samples passed QC, we found (100%) of our samples passed. Similar to previous reports, the values from the QC step was able to predict the ability of the samples to be restored successfully as evidenced by the pre vs. post-restore sample call rates for all samples (Fig. 1). We found that not only was the FFPE restore step successful for sheep samples, but it was required to obtain useable data from the OvineSNP50 BeadChip. All 48 samples pre-restore had call rates of zero, low average MAF, SNP call frequency, and GenTrain scores indicating unsuccessful genotyping of non-restored FFPE DNA using these methods. This result varies from reports of human FFPE samples using Illumina restore protocols and SNP arrays, where FFPE human samples have been genotyped without the restore step (Lips et al. 2005; Harada et al. 2011; Hosein et al. 2013; Hertz et al. 2015). The differences in these results could stem from greater annotation of the human genome, increased assay options available for human samples compared to sheep, the nature of the FFPE samples used here, and the lack of species-specific control metrics.

Comparison of pre vs. post-restore FFPE metrics show that restored samples yielded higher quality SNP data and overall assay metrics seen in Table 1. Illumina generated metrics including sample call rates, SNP average MAF, SNP call frequency, and GenTrain Scores used to assess assay quality were increased when the restoration step was used. Specifically, increases in sample call rates (0 to 0.88), and SNP call frequency (0.26 to 0.88) were found in post-restore samples. Our results align with increases in evaluated metrics in restored human FFPE samples, however we saw a more dramatic increase compared to pre-restore human samples. This difference could be due to the lack of pre-restore sample call rates (all 0) compared to studies using human FFPE samples where unrestored samples generated genotype calls (Hosein et al. 2013). This study did find increases in call rates post-restore, however the most improved sample went from (0.699–0.964) using the Illumina FFPE Restore and Illumina HumanCytoSNP FFPE array (Hosein et al. 2013). Further evaluation with larger sample sizes are needed to investigate this in the future.

Prior to comparing genotypes between matched post-restore FFPE and fresh DNA, both data sets underwent SNP and sample pruning to remove poor performing samples and SNPs. Post-restore FFPE samples with call rates < 80% were removed leaving 81% (39/48) to be used. To account for the FFPE nature of these sheep samples, an 80% sample call rate and SNP call frequency cutoff was used compared to 90% utilized in human FFPE samples in the Ilumina data sheet (Illumina). This cutoff was chosen based on successful human FFPE SNP analysis using this cutoff (Hertz et al. 2015). A total of 47,308 SNPs remained after pruning by SNP call frequency and average MAF 1% for post-restore FFPE samples. Compared to the post-restore, fresh DNA was pruned using a sample call rate and SNP call frequency cutoff of < 95% accounting for increased DNA quality in these samples compared to FFPE DNA. Studies using fresh human and ovine DNA for SNP analysis have reported success using this cutoff and was thus chosen for this analysis (Hertz et al. 2015; Brito et al. 2017b; Dovc et al. 2019). The same MAF cutoff of 1% was used to remove rare markers for both data sets. Together, data pruning left a total of 51,392 SNPs successfully genotyped in the fresh sample set, which is 4,084 SNPs more than the post-restore FFPE samples. This difference in valid SNPs for concordance analysis was expected as fresh DNA is higher quality due to lack of fixation induced DNA damage, and the higher number of total fresh samples available to be genotyped. We also found that despite restoration, p10GC sample scores and (unedited) FFPE sample call rates were lower compared to parallel fresh samples (Table 3). These values show that despite an increased validity in post-restored samples, FFPE DNA damage influences distribution of accurate GenTrain scores across all called genotypes for a sample.

**Table 3:** Sample Type Influence on p10 GC vs. Call Rate

Sample ID	Post-Restore FFPE p10GC	Post-Restore FFPE Call Rate	Fresh p10GC	Fresh Call Rate
1741	0.34	0.95	0.75	0.99
J-24	0.34	0.95	0.75	0.99
15111	0.34	0.95	0.75	0.99
4106	0.34	0.95	0.75	0.99

Data presented here was generated using GenomeStudio software (Illumina Inc). Values for the four individuals show significant increases in p10GC content when fresh samples were used compared to FFPE, as well as an increase in call rates. Although restoration yielded call rates close to fresh DNA, p10GC values were for post-restore FFPE were not increased to the same level as fresh DNA.

Our fresh sample data was compiled from 331 sheep after data pruning compared to the 39/48 FFPE samples passed for analysis, with four parallel samples used for direct genotype comparison. Despite the limited number of available parallel samples, post-restore FFPE allele calls were imported and compared to matched fresh sample allele calls across included SNPs. Using the GenomeStudio clustering algorithm and percent concordance calculation, over 99% of allele calls were found to be concordant between sample types, similar to FFPE vs. non-FFPE DNA reported in the literature (Thompson et al. 2005; Lips et al. 2005; Tuefferd et al. 2008; Hosein et al. 2013; Minca et al. 2014; Guo et al. 2019). Despite a varied number of matched FFPE vs. non-FFPE tissues and cancer tumor types investigated, overall high concordance was found in the literature ranging from 99% Lips et al. (Lips et al. 2005) in human colorectal tumors compared to matching DNA from whole blood, to 83% concordance reported from Tuefferd et al. (Tuefferd et al. 2008) using adenocarcinoma and matching fresh tissues. In contrast, Guo et al. (Guo et al. 2019) found poor concordance due to a batch effect resulting in only 20.8% concordance between FFPE and matched fresh-frozen samples using the Affymetrix platform. The influence of batch effect in this study illuminates the importance of experimental design to account for differences in sample types when conducting these investigations. Compared to our analysis of allele calls, these studies focused on tumor markers including copy number variations (CNVs) and regions of loss of heterozygosity (LOH).

Overall metrics show obvious differences due to sample type, however after pruning data, allele calls were found to be 99% concordant between restored FFPE and fresh samples from four sheep. Although only four samples had parallel DNA available, our results indicate successful genotyping of restored FFPE samples almost equal to fresh DNA in our study. The overlapping samples were fixed most recently in our study, thus concordance evaluation of FFPE samples fixed for various numbers of years with larger sample sizes is warranted. All included FFPE samples here were found to be successfully restored regardless of fixation age, but further evaluation is needed to verify this. Future work using non-human FFPE DNA for SNP microarray analysis should aim to validate microarray data using sequencing methods, and generate species-specific cutoff values to evaluate SNP array performance.

In conclusion, we have shown that the Illumina QC and FFPE restore workflow designed and tested for human samples, was successful on the sheep FFPE DNA tested here. The results indicated that this protocol was capable of restoring ovine FFPE DNA evident by increased sample call rates, SNP call frequencies, and overall assay metrics compared to pre-restore SNP data using the Illumina OvineSNP50 BeadChip. By directly comparing matched post-restore FFPE and fresh DNA we found 99% concordance between allele calls, although further validation using sheep FFPE samples from a variety of sources and other livestock species is needed. Our results indicate this may be a viable method to restore and utilize archived FFPE tissues of veterinary species, including cattle and pigs for which Illumina SNP BeadChips are available. Despite the growing focus on whole-genome sequencing (WGS) for genetic analysis studies, cost and practicality still inhibit widespread and international use in veterinary research. SNP arrays like those produced by Illumina, present a less expensive method for SNP genotyping, and thus a viable modality for international use. We believe the successful use of this methodology for a new sample type (FFPE) as described here has international implications for the progression of veterinary medicine. The future utilization and restoration of livestock FFPE DNA has the potential to aid to identify genetic markers for selective breeding programs for disease control, production traits, and enhancement of animal welfare (Rexroad et al. 2019; M Rosa et al. 2020; Thorne et al. 2021).

## Supplementary Information

Below is the link to the electronic supplementary material.

Supplementary file1 (DOCX 22 kb)

## Data Availability

All SNP assay results for FFPE samples and raw data are available upon request.
