# Supplementary material for: Successful restoration of archived ovine formalin fixed paraffin-embedded tissue DNA and single nucleotide polymorphism analysis
Source: Vet Res Commun. 2022 May 26;47(1):131–9. doi: 10.1007/s11259-022-09937-0 (PMC9873697; doi:10.1007/s11259-022-09937-0)
Supplement: Supplementary file 1 — Supplementary file1 (DOCX 22 kb) [file 11259_2022_9937_MOESM1_ESM.docx]

Supplementary Figure 1:

**FFPE Sample and Scroll Details:**

| **Sample ID** | **Sample Origin** | **Sex** | **Age at Necropsy** | **Breed** | **Age of FFPE Scroll** | **FFPE Tissue Type** |
| --- | --- | --- | --- | --- | --- | --- |
| C257 | CH | F | 6y, 9m | Kattupakkam | 7y | LIV |
| C168 | CH | F | 7y, 9m | Kattupakkam | 7y | LIV |
| C374 | CH | F | 5y, 10m | Kattupakkam | 7y | LIV |
| C495 | CH | F | 47, 9m | Kattupakkam | 7y | LIV |
| C424 | CH | F | 5y, 9m | Kattupakkam | 7y | LIV |
| C361 | CH | F | 6y, 6m | Kattupakkam | 7y | LIV |
| UMN1 | UNM | F | 4y | Unk | 7y | MLN |
| C696 | CH | F | 2y, 9m | Kattupakkam | 7y | LIV |
| C699 | CH | M | 2y,9m | Kattupakkam | 7y | LIV |
| JD184 | CH | F | 7y, 9m | Kattupakkam | 7y | LIV |
| JD254 | CH | F | 6y, 9m | Kattupakkam | 7y | LIV |
| JD522 | CH | F | 4y, 9m | Kattupakkam | 7y | LIV |
| JD660 | CH | F | 2y, 10m | Kattupakkam | 7y | LIV |
| JD629 | CH | F | 2y, 10m | Kattupakkam | 7y | LIV |
| JD656 | CH | F | 3y, 1m | Kattupakkam | 7y | LIV |
| JD783 | CH | M | 1y,9m | Kattupakkam | 7y | LIV |
| JD803 | CH | M | 1y,9m | Kattupakkam | 7y | LIV |
| JD738 | CH | F | 2y, 8m | Kattupakkam | 7y | LIV |
| MJD347 | CH | F | 6y,7m | Kattupakkam | 7y | LIV |
| MJD533 | CH | F | 4y,9m | Kattupakkam | 7y | LIV |
| MJD637 | CH | F | 3y,8m | Kattupakkam | 7y | LIV |
| MJD663 | CH | M | Unk | Kattupakkam | 7y | LIV |
| MJD826 | CH | M | 1y, 8m | Kattupakkam | 7y | LIV |
| MJD890 | CH | M | Unk | Kattupakkam | 7y | LIV |
| MJD790 | CH | M | 1y,9m | Kattupakkam | 7y | LIV |
| MJD741 | CH | F | 2y, 8m | Kattupakkam | 7y | LIV |
| MJD653 | CH | F | 3y,1m | Kattupakkam | 7y | LIV |
| M258 | CH | F | 6y, 9m | Kattupakkam | 7y | LIV |
| M375 | CH | F | 5y, 10m | Kattupakkam | 7y | LIV |
| M437 | CH | F | 5y, 9m | Kattupakkam | 7y | LIV |
| M510 | CH | F | 47, 9m | Kattupakkam | 7y | LIV |
| M581 | CH | F | 3y, 9m | Kattupakkam | 7y | LIV |
| M724 | CH | F | 2y,9 | Kattupakkam | 7y | LIV |
| M412 | CH | F | 5y,9m | Kattupakkam | 7y | LIV |
| M444 | CH | F | 5y,8m | Kattupakkam | 7y | LIV |
| M616 | CH | F | 3y, 9m | Kattupakkam | 7y | LIV |
| OKST6 | OKST | F | Unk | Unk | 8y | INT |
| OKST1 | OKST | F | Unk | Unk | 12y | INT |
| OKST2 | OKST | F | Unk | Unk | 7y | INT |
| UNB1 | UNB | M | Unk | Unk | Unk | LIV/SPL |
| UNB2 | UNB | M | Unk | Unk | Unk | LIV/SPL |
| UNB3 | UNB | F | Unk | Unk | Unk | LIV/SPL |
| UMN2 | UNM | F | 4y | Katahdin | 6y | MLN |
| UMN3 | UNM | F | 3y | Mixed | 4y | MLN |
| TR-15111 | TR | F | 4y | Royal White x White Dorper | 1y | LIV |
| TR-1741 | TR | F | 2y | Royal White | 1y | LIV |
| TR-J24 | TR | F | 2y | Royal White x White Dorper | 1y | LIV |
| TR-4106 | TR | F | 5y | White Dorper x Dorset | 1y | LIV |

**Supplementary Figure 1 Legend:** Information regarding sample origin, sex, age of animal at necropsy, breed, age of FFPE cassette, and tissue type included in FFPE scroll are provided. All FFPE samples were fixed using 10% buffered formalin and kept at room temperature (15-25°C) until use.

CH – Chennai Flock – Tamil Nadu Veterinary and Animal Sciences University

TR – University of Wisconsin Madison

OKST – Oklahoma State University

UNB – University of Nebraska

UNM- University of Mississippi

M- Male (ram)

F- Female (ewe)

Y – year

M- month

Unk – Unknown, information unavailable

INT– Intestine

LIV– Liver

LN – Lymph Node

LIV/SPL – Liver / Spleen

MLN – Mesenteric lymph node
